# WOMEN'S EXPERIENCES OF AGING AND SOCIAL CONNECTEDNESS

**DOI:** 10.1093/geroni/igz038.775

**Published:** 2019-11-08

**Authors:** Nicky J Newton, Jamila Bookwala

**Affiliations:** 1 Wilfrid Laurier University, Waterloo, Canada; 2 Lafayette College, Easton, Pennsylvania, United States

## Abstract

Models of aging, such as the successful aging framework outlined by Rowe & Kahn (1987; 2015) should be holistic, necessitating the inclusion of health, psychosocial factors, and social connectedness. Even at the oldest ages, life expectancy and rates of survival are increasing, yet these longer lives are accompanied with disease and disability, especially among women (Crimmins & Beltrán-Sánchez, 2010); thus, maximizing health and well-being during these post-retirement years, which can often span decades, is a high priority. However, models of age-related change, such as those relating to age-related transitions, are predominantly based on men’s experiences; less is known about how women navigate later life (Calasanti, 2010; Kim & Moen, 2002). The presentations in this symposium provide quantitative and qualitative data from women of a broad age range concerning their experiences of aging, with the shared theme of social relationships. Sherman examines the relationship between personality and social support for well-being outcomes in Native American, African American, and European American women (Mage = 57). Conceptualizing aging as the quintessential life transition, Newton outlines the diverse themes of physical, psychological, and social aging from interviews with older women (Mage = 72). Fuller and Toyama find that for older women (Mage = 80), friendships mattered more than family, and counting on neighbors could even be detrimental in terms of life satisfaction and stress. Taken together, these presentations provide a varied picture of what it means for women to ‘age well’, suggesting nuanced ways in which we might conceptualize theories of aging for women.

